# Exploring the presence of narcolepsy in patients with schizophrenia

**DOI:** 10.1186/s12888-016-0859-9

**Published:** 2016-06-01

**Authors:** Gemma Sansa, Alba Gavaldà, Carles Gaig, José Monreal, Guadalupe Ercilla, Roser Casamitjana, Gisela Ribera, Alex Iranzo, Joan Santamaria

**Affiliations:** Neurology Service and Multidisciplinary Sleep Disorders Unit, Hospital Parc Taulí, Sabadell, Spain; Neuropsychology Department, Neurology Service. Hospital Parc Taulí, Sabadell, Spain; Neurology Service and Multidisciplinary Sleep Disorders Unit, Hospital Clínic of Barcelona, Barcelona, Spain; Psychiatry Service, Hospital Parc Taulí, Sabadell, Spain; Immunology Laboratory, Hospital Clínic of Barcelona, Barcelona, Spain; Biochemistry and Molecular Genetics Laboratory, Centre de Diagnòstic Biomèdic (CDB), Hospital Clínic of Barcelona, Barcelona, Spain

**Keywords:** Hallucinations, Narcolepsy, Hypocretin, Psychotic disorders, Schizophrenia

## Abstract

**Background:**

There are several case reports of patients with narcolepsy and schizophrenia, but a systematic examination of the association of both disorders has not been done. The aim of this work is to assess the frequency of narcolepsy with cataplexy in a large consecutive series of adult patients with schizophrenia and schizoaffective disorder.

**Methods:**

We screened 366 consecutive patients with schizophrenia or schizoaffective disorder with a sleep questionnaire and the Epworth Sleepines scale (ESS) exploring narcoleptiform symptoms. Those who screened positive were assessed by a sleep specialist, and offered an HLA determination. CSF hypocretin-1 determination was proposed to those who were HLA DQB1*06:02 positive.

**Results:**

On the screening questionnaire, 17 patients had an ESS score ≥11 without cataplexy, 15 had cataplexy-like symptoms with an ESS score < 11, and four had an ESS score ≥11 plus cataplexy-like symptoms. Of those, 24 patients were evaluated by a sleep specialist. Five of these 24 were HLA DQB1*06:02 positive, and three of these five subjects underwent lumbar puncture showing normal hypocretin-1 levels.

**Conclusions:**

Our results suggest that narcolepsy with cataplexy is not an unrecognized disease in adult patients with schizophrenia or schizoaffective disorder.

**Electronic supplementary material:**

The online version of this article (doi:10.1186/s12888-016-0859-9) contains supplementary material, which is available to authorized users.

## Background

Narcolepsy with cataplexy (narcolepsy type 1) is a chronic neurological disease with an estimated lifetime prevalence of 2.5–5 per 10.000 people [1, 2]. Clinically, it is characterized by excessive daytime sleepiness (EDS), cataplexy, hypnagogic/hypnopompic hallucinations and sleep paralysis, as well as other sleep disturbances, such as sleep fragmentation [3]. Symptoms usually begin in adolescence, ^1,4^ and they are the result of hypocretin deficiency [4].

Hypocretins are neuropeptides that have a central role in the control of alertness [5, 6]. Undetectable or low hypocretin-1 levels in cerebrospinal fluid (CSF) are found in 95 % of narcoleptic patients with cataplexy [4, 7]. The mechanism causing this hypocretin deficit is still unknown, but is currently thought to be immune-mediated since up to 85–95 % of patients with narcolepsy with cataplexy have the HLA allele DQB1*06:02 [3, 8].

Hypocretin deficit is closely related to human leukocyte antigen (HLA) DQ B1*06:02 and cases of hypocretin-deficient narcolepsy without this HLA haplotype are exceptional. HLA typing is a useful screen before lumbar puncture since 98 % of patients with hypocretin-1 deficiency are DQB1*06:02 positive. ^7^ There are neurological disorders that can cause secondary narcolepsy [9, 10]. Clear-cut or definite cataplexy is considered a pathognomonic symptom for diagnosing narcolepsy [11] and is defined as more than one episode of generally brief and usually bilaterally symmetrical sudden loss of muscle tone with retained consciousness precipitated by strong emotions typically associated with laughter or elation. Some subjects may report features reminiscent of cataplexy that are not clear-cut, either because they are mild, atypical or only subjective experiences, a situation termed “cataplexy-like” particularly when using questionnaires in population-based studies [12, 13]. Narcolepsy type 1 [9] is diagnosed when hypersomnia is accompanied by 1) cataplexy and abnormal multiple sleep latency test (MSLT) or 2) low values of hypocretin-1 concentration in CSF.

Schizophrenia is one of the most devastating mental illnesses and has an estimated lifetime prevalence of 7, 2 per 1.000 people [14]. The core symptoms of schizophrenia include delusions, hallucinations and thought disorder [15]. Schizophrenic symptoms usually also emerge during adolescence [15, 16]. Dopamine neurotransmission abnormalities in the mesocorticolimbic system with an increase in dopamine synthesis, dopamine release and resting-state synaptic dopamine concentrations play a central role in the generation of schizophrenic symptoms [17, 18]. The etiology of schizophrenia is unknown, although and interplay between genetic and environmental factors has been suggested [15].

There are several previous case reports of narcoleptic patients with psychotic symptoms [19, 20] and of schizophrenic patients with narcoleptic features [21–23]. Two recent studies have evaluated the presence of schizophrenia in a large number of children and adolescents with narcolepsy [24, 25], finding a small group of individuals that developed schizophrenia after being diagnosed with narcolepsy.

Several explanations could account for a possible association between narcolepsy and schizophrenia. Psychosis may develop in narcoleptic patients as a consequence of stimulant therapy, although this side effect seems infrequent even in patients treated with high doses of stimulants over a prolonged period time [23, 26]. Another option is that some narcoleptic symptoms, especially hypnagogic and hypnopompic hallucinations, could be misdiagnosed as an active psychotic state of schizophrenia [22, 23, 27]. A third option is that narcolepsy and schizophrenia could occur in the same patient because both diseases might share similar physiopathological factors. Finally, the coincidental occurrence of schizophrenia and narcolepsy in the same patient could also occur by chance. The frequency of coexistent schizophrenia and narcolepsy could be expected to be 18–36 cases in a population of ten million based on the independent prevalence of these two diseases. It is possible, however, that patients with schizophrenia who develop narcolepsy could be unrecognized because sleepiness could be attributed to the antipsychotic treatment and cataplexy could be alleviated or masked by the same antidopaminergic treatment [28].

The aim of this work was to assess the frequency of undiagnosed narcolepsy in a large cohort of patients with schizophrenia. Finding some patients with narcolepsy in our patients could support a possible association between narcolepsy and schizophrenia would increase our understanding in both disorders.

## Methods

All consecutive patients with schizophrenia or schizoaffective disorder [29] followed at the adult Psychiatry Department of the Hospital Parc Taulí were proposed to participate in the study during their routine visits from February to June of 2013. Patients older than 18 years and able to answer the questions were invited to participate. Patients under 18 years were not included as they are controlled in another Psychiatric Department at our institution. The Psychiatric Ambulatory Unit of the Hospital Parc Taulí is the reference center for an approximate population of 280000 and consists of 4 psychiatric nurses and 13 psychiatrists, which during the period of the study visited approximately 600 patients with schizophrenia and schizoaffective disorder. Once the study protocol was approved it was explained to all the members of the unit and the psychologist of the study spent several days a week during the study period in the unit, reminding all the staff to invite patients to participate in the protocol. Psychiatrists and psychiatric nurses only considered eligible for the study those patients who –in their opinion- were able to answer the questions. Only seven patients were excluded later because we found their answers unreliable.

To assess whether narcolepsy was present in schizophrenia, we used a stepwise approach with three phases of evaluation.

In a first phase, consecutive patients were screened with a semi-structured questionnaire (Additional file 1) where the four main symptoms of narcolepsy were included, namely EDS, cataplexy, hypnagogic/hypnopompic hallucinations and sleep paralysis. This questionnaire was administered by a psychologist (AG) specially trained in sleep disorders. All interviews were conducted by the same psychologist, which had been specially trained in the symptoms of narcolepsy and had also clinical experience with schizophrenia. The presence of cataplexy was explored using the following question: “have you ever felt sudden weakness of your muscles when you experience a strong emotion?”. Sleep paralysis was assessed asking: “have you ever felt unable to move for a few moments as if you were paralyzed, just after waking up or when falling asleep despite being awake?” Hypnagogic/hypnopompic hallucinations were explored asking “have you ever noticed or seen things or people that do not actually exist, just after waking up or when falling asleep?”; patients that responded positively were asked to explain what exactly they felt in those moments and try to differentiate these hallucinations from schizophrenic hallucinations that could appear during wakefulness. Finally the presence of EDS was assessed with the Epworth sleepiness scale (ESS) and an score ≥ 11 was considered indicative of this symptom [30]. When patients were unable to answer a particular question in the Epworth Sleepiness Scale (for instance in a car, while stopped for a few minutes in the traffic), it was excluded in the total ESS count (meaning that maximum possible punctuation in that particular case was 21 instead of 24, and the resulting score was adjusted for this modification). Data on age, gender and age at onset of schizophrenia were also collected.

In a second phase of the study, patients with an ESS score ≥ 11 and/or cataplectic-like features were reevaluated by a neurologist from the Multidisciplinary Sleep Unit of the Hospital Parc Taulí (GS) who conducted a systematized clinical interview focusing on the presence and features of EDS, cataplexy, hypnagogic/hypnopompic hallucinations and sleep paralysis.. Patients who did not confirm cataplexy-like symptoms in this second interview were excluded. In addition, current medications were evaluated as well as the existence of other sleep disturbances (sleep fragmentation, symptoms suggestive of obstructive sleep apnea (OSA), insufficient sleep, and cyrcadian rhythm disorder). In those patients in whom EDS and/or cataplexy experiences were considered to be likely present, HLA typing was tested.

As medications such as antidepressants or other psychotropic drugs may significantly affect rapid eye movement (REM) sleep, it is recommended that polisomnography and multiple latency sleep test should be used to diagnose narcolepsy only in patients free of drugs that influence sleep. Drugs must be stopped for at least 14 days (or at least five times the half-life of the drug and longer-active metabolite), confirmed by a urine drug screen [9].

Since psychotropic medications could not be stopped in our patients for clinical management and ethical reasons we could not use these objective tests for the diagnosis of narcolepsy. However, given that narcolepsy-cataplexy type 1 occurs almost exclusively in patients with HLA DQB1*06:02, we tested the presence of this HLA haplotype in patients with symptoms suggestive of narcolepsy.

In a third phase, hypocretin-1 level determination in CSF was proposed to those patients who were HLA DQB1*06:02 positive. If a patient (and caregiver when necessary) accepted, 5 ml of CSF were obtained and immediately stored at −80 °C. The lumbar puncture was performed in the morning, before 11 am. Hypocretin-1 levels in CSF were determined using commercially available direct radio-immunoassay kit (Phoenix Pharmaceuticals, USA) as previously described [4, 31, 32] at the Hospital Clinic of Barcelona. In order to minimize inter-assay variation, reference samples with internal controls with known hypocretin-1 values were included in each assay and the values were adjusted accordingly as recommended [33].

## Results

We included 366 consecutive patients with the diagnosis of schizophrenia or schizoaffective disorder (Table 1). There were 226 men and 140 women, with a mean age of 44.3 ± 12 (range, 18–81) years and a mean age at diagnosis of the psychiatric disorder of 25.3 ± 8.1 (range, 7–56) years.

**Table 1:** DSM-IV-TR diagnosis

Diagnosis	DSM-IV-TR diagnosis code	Number of patients (*n* = 366)
Schizophrenia, Disorganized Type	295.10	13 (3.5 %)
Schizophrenia, Paranoid Type	295.30	240 (65.6 %)
Schizophrenia, Residual Type	295.60	27 (7.4 %)
Schizophrenia, Undifferentiated Type	295.90	17 (4.6 %)
Schizoaffective Disorder	295.70	69 (18.9 %)

Sixty five percent of the patients were diagnosed as paranoid type of schizophrenia, being schizoaffective disorder diagnosis the second more frequent one

**Fig. 1:**
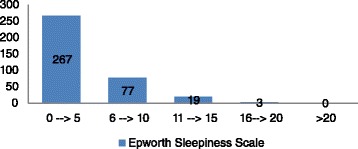
Epworth Sleepiness Scale values. Distribution of Epworth Sleepiness Scale scores in the 366 patients screened are shown in the following figure

The mean ESS score was 4.1 ± 3.4 (range 0–18). Twenty-two (6 %) patients had an ESS ≥11 (Fig. 1).

Nineteen (5.1 %) patients reported cataplexy-like experiences. Thirty-three (9 %) responded that they presented sleep paralysis and 78 (21.3 %) hypnagogic/hypnopompic hallucinations.

**Fig. 2:**
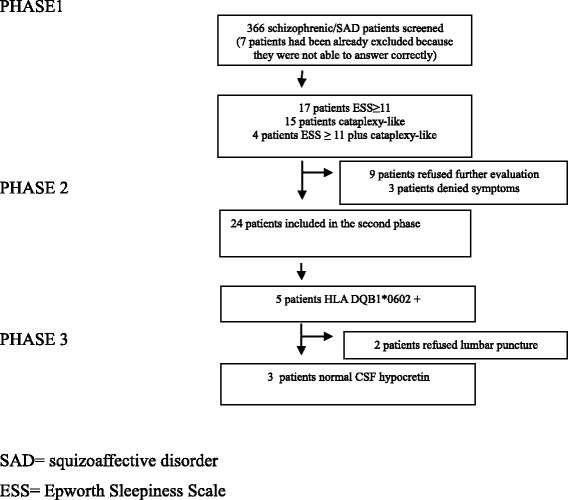
Flow-chart describing the study results. Of the 366 patients screened, 35 presented hypersomnia and/or cataplexy. Of them, 5 were HLA positive and no abnormal hypocretin value was obtained

Thirty-six (9.8 %) patients had an ESS score ≥ 11 and/or responded affirmatively to the cataplexy question (Fig. 2). Seventeen had an ESS score ≥ 11 without cataplexy, 15 presented cataplexy-like symptoms with an ESS score < 10, and 4 patients had an ESS score ≥ 11 and/or responded affirmatively to the cataplexy question. All these 36 patients were offered to participate in the second phase, and 27 accepted. Three patients were excluded after detailed questioning by the sleep specialist (GS) who considered that neither EDS nor cataplexy-like experiences did occur. Twenty-four patients were finally included in this second phase, 11 women and 13 men, with a mean age of 39.7 ± 10 (range, 21–57) years, and a mean age at onset of the psychiatric disease of 22.8 ± 7.5 (range, 12–44) years. Clinical characteristics of this group of patients are detailed in Table 2. In 14 patients EDS was temporally related to the beginning of the antipsychotic treatment. Six out of these 24 patients reported features reminiscent of cataplexy, but no case of clear-cut cataplexy was found. Five patients presented hallucinations mainly during sleep-wake transition (two auditory –knock on the wall, a buzz-, one visual –could not specify which- and two tactile –both being touched-). Sleep paralysis was present in five out of the 24 patients.

**Table 2:** Clinical characteristics of the patients. Characteristics of the patients included in the second phase (ESS ≥ 11 and/or cataplexy) are summarized in the following table

	Patients (*n* = 24)
Age (years)	39.7 ± 10.3 (range 21–57)
Age of onset psychiatric disorder (years)	22.8 ± 7.5 (range, 12–44)
Male gender (%)	54.2
Body Mass Index (n)	32.8 ± 5.6 (range, 20–43.7)
Epworth Sleepiness Scale score (n)	11.4 ± 4.1 (range, 2–18)
Duration of EDS (years)	16.8 ± 14.2 (range, 1–50)
Possible cataplexy (n/%)	6 (25)
Hypnagogic/hypnopompic Hallucinations (n/%)	5 (20.8)
Sleep paralysis (n/%)	5 (20.8)
Estimated Nocturnal sleep amount (hours)	9.7 ± 2 (range, 5–13.5) (14 patients slept ≥ 10 h/night)
Nocturnal sleep fragmentation (≥2 awakenings/night) (%)	1 (4.1)
Refreshing short naps (%)	0 (0)
Circadian rhythm disorder	1 patient had advanced sleep phase disorder 4 patients had delayed sleep phase disorder
Snorers (n/%)	12 (50)
Witnessed apneas (n/%)	6 (25)
Previous diagnosis of OSAS (n/%)	1 (4.1)
Neuroleptic treatment (%)	100
Benzodiazepine treatment (%)	37.5
Antidepressant treatment (%)	45.8
Antiepileptic treatment (%)	54.2

*EDS* Excessive daytime sleepiness, *OSAS* Obstructive sleep apnea syndrome

Twelve (50 %) patients were snorers and in six of them their bed partners reported apneas during sleep. Only one patient had been previously diagnosed of obstructive sleep apnea syndrome with a sleep study, which showed an apnea/hypopnea index of 78, but she did not tolerate continuous positive airway pressure treatment. Nine patients were treated with one neuroleptic drug, 13 patients with two, and three patients with three. Five patients were treated with one benzodiazepine, and four with two. Nine patients were treated with one antidepressant drug, one patient with two, and one patient with three. Eight patients were treated with one antiepileptic drug, one patient with two and one patient with three.

Five out of the 24 patients had HLA DQB1*06:02, none of them homozygote nor positive for DQB1*03. Clinical characteristics of these five patients are shown in Table 3. In these 5 patients a lumbar puncture to measure CSF hypocretin-1 was proposed. Patient 1 and 2 refused. Hypocretin-1 levels were finally determined in three patients showing normal values (>200 pg/ml) in all of them.

**Table 3:** Clinical characteristics of HLA positive patients. The clinical characteristics of the five HLA positive patients with hypersomnia and/or cataplexy are described in this table

Patient	1*	2*	3*	4*	5**
Gender	F	M	M	F	F
Age at onset of psychiatric disorder (years)	29	12	15	18	19
Age at onset of hypersomnia (years)	29	12	6	18	19
Epworth Sleepiness Score (n)	12	14	14	16	10
Cataplexy present	NO	NO	NO	YES	YES
Sleep paralysis present	NO	YES	YES	NO	NO
Hypnagogic/hymnopoimpic hallucinations present	NO	NO	NO	YES	YES
BMI (n)	31.2	43.7	26	37.1	20
Snoring present	NO	YES	YES	NO	NO
Witnessed apneas	NO	YES	YES	NO	NO
Mean sleep at nighttime (hours)	8	8	7.5	13	10.5
Hypocretin-1 value (pg/ml)	NA	NA	276.5	333.7	419.8

*BMI* body mass index, *NA* not available

* DSM Diagnosis was 295.30 ** DSM Diagnosis was 295.90

## Discussion

To our knowledge, this is the first study evaluating systematically and prospectively the prevalence of narcolepsy type 1 in a large group of patients with schizophrenia or schizoaffective disorder. Since antidepressants or other psychotropic drugs may significantly affect REM sleep, we did not use polysomnography and multiple latency sleep to diagnose narcolepsy in our patients, given that these drugs could not be stopped for a long enough period of time. We used instead a stepwise approach, with an initial screening of characteristic symptoms of narcolepsy in 366 patients and offering HLA determination in those who referred hypersomnia and/or possible cataplexy. Last step was determining hypocretin in CSF in those who were HLA DQB*06:02 positive and had EDS and/or cataplexy-like experiences. We did not find any patient with clear narcolepsy type 1 in our cohort. Although we only measured CSF hypocretin in three patients, they were the majority of subjects reporting hypersomnia and cataplexy-like symptoms who had a positive HLA DQB1*06:02. Although there are cases of DQB1*06:02 negative patients with narcolepsy type 1, this is highly infrequent since it is estimated that less than one in 500 HLA-negative patients will have low CSF hypocretin-1 levels [34]. Since the poblational prevalence of narcolepsy is between 2.5 and 5 per 10000 inhabitants, it would be required to include between 4604 and 9209 patients to have a 90 % chance of observing 1 case. If schizophrenia or schizoaffective disorders were associated to an increased prevalence by 10 fold, the numbers required to detect one case would be between 459 and 919. A small sample confidence interval for the estimate, based on the exact probabilities of the binomial distribution, would be between 0 and 1.003 %, so that in our sample a prevalence of narcolepsy 1 % or lower (i e: increased by 20 to 40 fold) could not be excluded.

It is considered that sleep disturbances, particularly poor sleep quality, are common in schizophrenia [35]. Although hypersomnia is a common side effect of neuroleptics, few studies have focused on this aspect. While some authors [36] found a mean sleep latency >36 % longer (sleep propensity was lower) in 30 untreated patients with schizophrenia than in healthy subjects, other authors [37] reported a prevalence of hypersomnia of 24–31 % in 1493 treated schizophrenic patients. In our sample the frequency of hypersomnia was remarkably lower and only 5.7 % of the patients had an ESS ≥ 11, usually temporary related to the beginning of the treatment for the psychiatric disorder or the coexistence of a possible sleep breathing disorder, as in our cohort half of them were snorers, and the mean body mass index was 32.8 (moderately obese). The lower prevalence of hypersomnia in our study could also be due to the long duration of therapy and disease compared to the previous studies [36, 37].

Our findings do not suggest that in a large, single-center sample of adult schizophrenic patients narcolepsy is underdiagnosed. However, it is possible that the association of schizophrenia with narcolepsy could be age dependent, with higher risk of developing schizophrenia in only those patients who had previously initiated narcolepsy in childhood or early adolescence. Huang [25] reported that subjects with narcolepsy linked to schizophrenia were younger than those narcoleptics without schizophrenia (mean age of onset of narcolepsy, 11.25 ± 3.92 years vs 12.59 ± 3.41 years). Canellas ^24^ reported that the age of onset of EDS was 12.6 ± 2.3 year, age at narcolepsy diagnosis was 17.9 ± 6.2 years and age at onset of psychotic symptoms was 16.9 ± 3.4 years. Since in our sample, mean age at diagnosis of schizophrenia and schizoaffective disorder was higher, with a mean age of 25 years, we cannot completely exclude that a relationship between narcolepsy and schizophrenia exists only in younger patients.

Our study has limitations such as the lack of inclusion of patients who were considered unable to answer the questionnaires before the screening, the lack of a detailed expert sleep history in all the 336 patients initially evaluated, the lack of sleep recordings, and the lack of HLA and hypocretin-1 determinations in the whole cohort. However, in the majority of patients reporting EDS and narcoleptiform symptoms in the screening evaluation the sleep expert confirmed these symptoms. On the other hand, sleep recordings are less useful in patients treated with psychotropic drugs and the determination of CSF hypocretin-1 in such a large sample of patients with schizophrenia is almost unfeasible.

## Conclusions

Based in our findings, narcolepsy is not an underrecognized disease in adult patients with schizophrenia or schizoaffective disorder. Complaints of hypersomnia are rare in these patients and whereas hypnagogic/hypnopompic hallucinations are the most frequent narcoleptiform symptom.

### Ethics aproval

The study was approved by ethics committees of both hospitals (Hospital Parc Taulí and Hospital Clínic), and a written informed consent of patients was obtained.

### Consent for publication

Not applicable.

### Availability of data and materials

The data will not be shared because it contains indirect identifying data.

## Additional file

Additional file 1: Screening questionnaires: This questionnaire was developed as a screening tool for the main symptoms of narcolepsy. (DOCX 13 kb)

## Abbreviations

CSF: cerebrospinal fluid
EDS: excessive daytime sleepiness
ESS: Epworth sleepines scale
HLA: human leukocyte antigen
MSLT: multiple sleep latency test
OSA: obstructive sleep apnea
REM: rapid eye movement
