# Supplementary material for: Exploring the presence of narcolepsy in patients with schizophrenia
Source: BMC Psychiatry. 2016 Jun 1;16:177. doi: 10.1186/s12888-016-0859-9 (PMC4888670; doi:10.1186/s12888-016-0859-9)
Supplement: Additional file 1: — Screening questionnaires: This questionnaire was developed as a screening tool for the main symptoms of narcolepsy. (DOCX 13 kb) [file 12888_2016_859_MOESM1_ESM.docx]

**NARCOLEPSY IN PATIENTS WITH SCHIZOPHRENIA**

**Patient name:________________________________________________**

**Date of birth:____/____/______ Gender: Male Female**

**Identification number: ________________ Study code: MC-SCZ-_______**

**Date:___/___/______ Age:______**

**Age at diagnosis of psychiatric disease: ______**

**Psychiatric disorder: ______**

Cataplexy: “have you ever felt sudden weakness of your muscles when you experience a strong emotion?” Yes /No

Sleep paralysis: “have you ever felt unable to move for a few moments as if you were paralyzed, just after waking up or when falling asleep despite being awake?” Yes /No

Hypnagogic/hypnopompic hallucinations: “have you ever noticed or seen things or people that do not actually exist, just after waking up or when falling asleep?” Yes /No

Epworth Sleepiness Scale:

Use the following scale to choose the most appropriate number for each situation:

0 = would never doze

1 = slight chance of dozing

2 = moderate chance of dozing

3 = high chance of dozing

Situation Chance of Dozing (0-3)

Sitting and reading ________________________________________

Watching TV ________________________________________

Sitting, inactive in a public place (e.g. a theatre or a meeting) _________

As a passenger in a car for an hour without a break _________________

Lying down to rest in the afternoon when circumstances permit ________

Sitting and talking to someone __________________________________

Sitting quietly after a lunch without alcohol ________________________

In a car, while stopped for a few minutes in the traffic ________________
